# New benzimidazole derivative compounds with in vitro fasciolicidal properties

**DOI:** 10.1186/s13071-024-06224-6

**Published:** 2024-04-03

**Authors:** Elora Valderas-García, Verónica Castilla-Gómez de Agüero, Laura González del Palacio, Giulio Galli, Nerea Escala, Marta Ruiz-Somacarrera, Marta González-Warleta, Esther del Olmo, Rafael Balaña-Fouce, María Martínez-Valladares

**Affiliations:** 1 Departamento Sanidad Animal, Instituto de Ganadería de Montaña, CSIC-Universidad de León, Grulleros, 24346 León, Spain; 2 Departamento de Sanidad Animal, Facultad de Veterinaria, Universidad de León, Campus de Vegazana S/N, 24071 León, Spain; 3 Departamento de Ciencias Biomédicas, Facultad de Veterinaria, Universidad de León, Campus de Vegazana S/N, 24071 León, Spain; 4 Departamento de Ciencias Farmacéuticas: Química Farmacéutica, Facultad de Farmacia, Universidad de Salamanca, CIETUS, IBSAL, 37007 Salamanca, Spain; 5 Laboratorio de Parasitología, Centro de Investigacións Agrarias de Mabegondo, AGACAL-Xunta de Galicia, Abegondo, 15318 A Coruña, Spain; 6 Departamento de Ciencias Biomédicas, Institute of Biomedicine (IBIOMED), Universidad de León, 24071 León, Spain

**Keywords:** *Fasciola hepatica*, Benzimidazole, Anthelmintic resistance, Zoonotic disease

## Abstract

**Background:**

Control of the zoonotic food-borne parasite *Fasciola hepatica* remains a major challenge in humans and livestock. It is estimated that annual economic losses due to fasciolosis can reach US$3.2 billion in agriculture and livestock. Moreover, the wide distribution of drug-resistant parasite populations and the absence of a vaccine threaten sustainable control, reinforcing the need for novel flukicides.

**Methods:**

The present work analyses the flukicidal activity of a total of 70 benzimidazole derivatives on different stages of *F. hepatica*. With the aim to select the most potent ones, and screenings were first performed on eggs at decreasing concentrations ranging from 50 to 5 µM and then on adult worms at 10 µM. Only the most effective compounds were also evaluated using a resistant isolate of the parasite.

**Results:**

After the first screenings at 50 and 10 µM, four hit compounds (BZD31, BZD46, BZD56, and BZD59) were selected and progressed to the next assays. At 5 µM, all hit compounds showed ovicidal activities higher than 71% on the susceptible isolate, but only BZD31 remained considerably active (53%) when they were tested on an albendazol-resistant isolate, even with values superior to the reference drug, albendazole sulfoxide. On the other hand, BZD59 displayed a high motility inhibition when tested on adult worms from an albendazole-resistant isolate after 72 h of incubation.

**Conclusions:**

BZD31 and BZD59 compounds could be promising candidates for the development of fasciolicidal compounds or as starting point for the new synthesis of structure-related compounds.

**Graphical Abstract:**

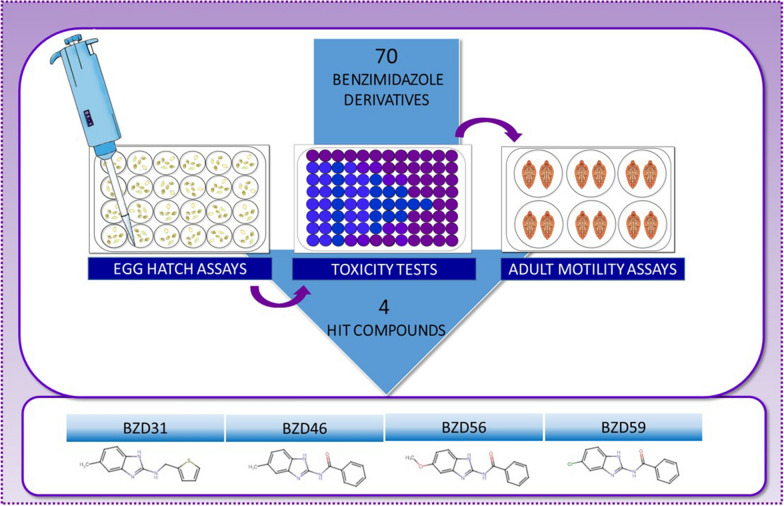

**Supplementary Information:**

The online version contains supplementary material available at 10.1186/s13071-024-06224-6.

## Background

Fasciolosis is a highly pathogenic foodborne disease affecting both humans and livestock, resulting from infection by the trematodes *Fasciola hepatica* and *Fasciola gigantica*, commonly referred to as liver flukes [1]. Different studies have documented that *F. hepatica* exhibits the widest geographical distribution among helminth parasites, being present on every continent except Antarctica [2], with a predominance in temperate regions. In contrast, *F. gigantica* primarily inhabits tropical regions in Africa and Asia [3]. However, the occurrence of hybrids is plausible, producing intermediate forms in areas where the two species overlap [4].

These parasites have an indirect life cycle involving an invertebrate (*Lymnaea* spp.) and a vertebrate host, which includes large domestic and wild ruminant species, such as sheep, cattle, and goats [5]. In livestock, its significance lies mainly in the economic costs of infection. Subclinical losses contribute substantially to these costs and are estimated to reach approximately US$3.2 billion per year [5], impacting upon animal health and food safety. Chronic infections are highly prevalent and frequently result in reduced fertility, milk production, growth rates, and feed conversion efficiency [6, 7].

In terms of human infection, the World Health Organization (WHO) anticipated that 180 million people are at risk of infection, and 2.4 million are infected [5]. In addition, fasciolasis is considered a neglected tropical disease, constituting a serious public health issue due to the long-term chronic and debilitating nature of the infection and secondary complications, such as cholangiocellular carcinoma [4], fibrosis, and cirrhosis [8].

Control of liver fluke relies on the use of anthelmintic drugs, with triclabendazole (TCBZ) being the most widely fasciolicidal drug used in animals and humans nowadays [9, 10] and the only one recommended by the WHO and the Pan American Health Organization (PAHO) for humans [11]. On the other hand, the range of treatments for livestock is wider, although with variations in commercial availability between countries. These treatments include benzimidazole (BZ) compounds such as albendazole (ABZ) and netobimin, as well as others, such as closantel, clorsulon, nitroxinil, and rafoxanide. All of them are known to be effective against adult worms, whereas TCBZ is the only one that also exhibits activity against juvenile stages (< 8 weeks) [12].

Despite decades of successful efficacy, their irresponsible and excessive utilization over time has led to the emergence of resistant parasite populations. More precisely, TCBZ failure in livestock is now widespread across all continents. Additionally, recent observations indicate the presence of resistance to other drugs, such as closantel, albendazol, and clorsulon [12–17]. Likewise, cases of human resistance to TCBZ have arisen in the last few years, heightening the level of concern [9, 18, 19].

Given the widespread resistance and the absence of an available vaccine, there is an urgent need for the discovery of new effective fasciolicidal drugs.

In this scenario, the present study assessed the in vitro activity of a series of new benzimidazole derivatives (BZDs) against several stages of the trematode *F. hepatica*. Some of the tested compounds had previously demonstrated in vitro and in vivo activity against two gastrointestinal nematodes infecting ruminants: *Teladorsagia circumcincta* and *Haemonchus contortus* [20–22].

## Methods

### Compounds

The BZDs tested in the presented study belonged to two different families: 2-(aryl)-benzimidazole (type I) and 2-amino benzimidazole (type II), which includes three sorts of structures: 2-arylsulfamido benzimidazoles (BZD25 and BZD26), 2-arylmethylamino benzimidazoles (BZD27 and BZD45), and 2-arylcarboxamido benzimidazoles (BZD46 and BZD70). These compounds were synthesized by the Department of Pharmaceutical Sciences of the University of Salamanca (Spain). All procedures were described in previous studies [20, 21].

Stock solutions of these compounds were prepared in dimethyl sulfoxide (DMSO, ≥ 99.9%, Sigma-Aldrich^®^, Spain). ABZ (≥ 99% purity, Sigma-Aldrich^®^, Spain), oxfendazole (OXF, ≥ 99% purity, Sigma-Aldrich^®^, Spain), ABZ sulfoxide (ABZSO, ≥ 99% purity, Sigma-Aldrich^®^, Spain), and TCBZ (≥ 99% purity, Sigma-Aldrich^®^, Spain) were also dissolved in DMSO.

### *Fasciola hepatica* isolates

#### Susceptible isolates

Eggs were recovered from gallbladders of cattle naturally infected with *F. hepatica* at the municipal slaughterhouse of León (Spain). For this, the bile of the gallbladders was filtered through sieves of different diameter size pores (500, 150, and 20 µm) using pressurized tap water. Eggs retained on the 20 µm pore size sieve were collected and stored at 4 °C in darkness until required within a maximum period of 2 months.

The susceptibility of the isolates to ABZ was confirmed by the egg hatch test (EHT) using an ABZ discriminant dose (DD) of 0.5 µM [23]. The technique is described in more detail below.

#### Resistant isolate

*F. hepatica* eggs were recovered after washing the gallbladder of three naturally infected sheep belonging to a flock suspected to be resistant to ABZ. From these eggs, metacercariae were produced at the Centro de Investigaciones de Mabegondo (AGACAL, Xunta de Galicia, Spain) following a previous protocol [24]. These metacercariae were used to infect eight sheep with a dose of 200 metacercariae. The infection was confirmed individually 3 months after the infection by coprological analysis using a sedimentation technique. At that point, sheep were randomly located in two groups of four animals: one treated with a therapeutic dose of ABZ (7.5 mg/kg) and the other one only with water, as control group. To assess the efficacy of ABZ in this isolate, all sheep were humanely euthanized by an intravenous administration of a lethal dose of sodium pentobarbital (Dolethal^®^, Vetoquinol, Spain) 2 weeks later for fluke recovery and counting. Afterwards, the in vivo efficacy of ABZ was calculated as the percentage of adult worm reduction compared with the untreated control. Additionally, the number of eggs per gram of feces (EPG) was estimated on the treatment day (mentioned as day 0) and 14 days later to determine drug efficacy by means of the fecal egg count reduction test (FECRT).

During the necropsy, gallbladders from sheep belonging to the untreated group were taken to collect the eggs and carry out the in vitro EHT, while flukes collected from livers were used to conduct the adult viability test (AVT).

The percentage of in vivo efficacy of ABZ and the FECRT were calculated using the following formulas, respectively:

$$\% {\text{ of Worm Reduction}} = { 1}00 \, \times \, \left( {{\text{Mean of adult worms in control group }}{-}{\text{ Mean of adult worms in treated group}}} \right)/\left( {\text{Mean of adult worms in control group}} \right).$$

$$\% {\text{ FECRT }} = { 1}00 \, \times \, \left( {{\text{Arithmetic mean EPG day }}0 \, {-}{\text{ Arithmetic mean EPG day 14}}} \right)/\left( {{\text{Arithmetic mean EPG day }}0} \right).$$

### Compound screening

The in vitro anthelmintic activity of a total of 70 BZDs was initially tested on *F. hepatica* eggs recovered from gallbladders of cattle naturally infected with ABZ-susceptible isolates. The first screening was performed using a fixed concentration of 50 µM. A cutoff value was set to continue the experiments with the most potent compounds. Therefore, only BZDs with ovicidal activities over 80% progressed to the next assays at 10 µM. In the same way, a third test was carried out with the most effective ones at 5 µM. At this last concentration, the ovicidal effect of ABZ, ABZSO, and OXF was also evaluated.

Those BZDs with an activity higher than 80% at a concentration of 10 µM were also screened on eggs and adults from the ABZ resistant isolate.

#### Egg hatch test (EHT)

The in vitro EHT described in the current study was based on previous studies [23, 25]. Briefly, 100–120 eggs were incubated in 990 µL of water and 10 μL of each working solution at 25 °C in the dark for a period of 12 h. After incubation, eggs were gently washed with tap water three times to facilitate compound removal and kept in darkness at 25 °C for 14 days. After this period, eggs were exposed to light for 2 h to stimulate the hatching of eggs. Then, hatched and unhatched eggs were evaluated and counted using an optical microscope. The term “hatched eggs” includes both hatched and embryonated eggs. Negative control eggs were incubated with 10 μL of DMSO in 990 μL of water, reaching a maximum DMSO concentration of 1% (v/v), while ABZ at 0.5 µM was used as positive control. Each compound at a specific concentration was tested in triplicate in the same EHT and repeated at least two different days.

With the aim to characterize the susceptibility of isolates from naturally infected cattle, the drug used in the EHT was ABZ, tested at a discriminant dose of 0.5 μM. Drug susceptibility is assumed when the ovicidal activity is over 70%, resistance when is lower than 40%, and a suspicion of resistance when activity is between these values at 0.5 μM.

The efficacy of each compound and drug was calculated as its ovicidal activity following the formula below:

$${\text{Ovicidal activity }}\left( \% \right) \, = { 1}00 \, \times \, \left( {\% {\text{ eggs hatched in negative control }} - \, \% {\text{ egg hatched after drug incubation}}} \right)/\left( {\% {\text{ egg hatched in control}}} \right).$$

#### Adult viability test (AVT)

The adult viability test was based on a previous study carried out by Kirchhofer et al. [26] with minor modifications. In brief, adult *F. hepatica* flukes were recovered from bile ducts of infected sheep with the resistant isolate. Flukes were quickly washed with 0.9% (w/v) NaCl and placed in six-well plates (Costar, Spain) with RPMI 1640 culture medium (Sigma-Aldrich^®^, Spain) supplemented with antibiotics (50 μg/mL streptomycin and 50 IU/mL penicillin; Sigma-Aldrich^®^, Spain) and 80 μg/mL of a hemin solution at 37 °C.

To monitor the anthelmintic effect of BZDs and the commercial drug ABZ, two flukes per well were incubated up to 72 h at a final concentration of 10 µM. DMSO at 0.5% was used as negative control. The individual movement of each fluke was examined using an inverted microscope at 24, 48, and 72 h. The activity of the compounds was estimated as motility inhibition, using a scale of fluke viability ranging from 3 (normal movements) to 0 (death, no movement observed for 2 min). All tests were repeated at least twice at different days.

Additionally, the percentage of motility inhibition for each compound was calculated from time 0 to 48 and 72 h after treatment using the following formula: 100 × (mean of the motility score of BZD at 24 h − mean of the motility score of BZD at 48 or 72 h)/(mean of the motility score of BZD at 24 h).

#### Cytotoxicity assays

The effect of the BZDs on cell viability was assessed on a human hepatocarcinoma HepG2 (ATCC^®^ HB-8065™, USA) cell line. Only compounds screened at a concentration of 10 µM were evaluated. The detailed protocol is described in a previous study carried out by our research group [20]. Briefly, 10,000 cells were seeded on 96-well plates and incubated with different concentrations of the compounds ranging from 1 to 100 µM at 37 °C and 5% CO_2_. After 72 h of exposure, viability of the cells was assessed using the alamarBlue (Fisher Scientific^®^, Spain) staining method according to the manufacturer’s recommendations.

#### Intestinal tolerability assays on murine intestinal organoids

The murine intestinal organoid cultures were prepared following Stemcell Technologies™ protocols (https://www.stemcell.com/intestinal-epithelial-organoid-culture-with-intesticult-organoid-growth-medium-mouse-lp.html#protocols). Small intestine sections from C57BL/6 mice were excised, washed with ice-cold PBS, minced, and 2 mm segments were washed extensively. The segments were treated with Gentle Cell Dissociation Reagent (Stemcell Technologies™, Canada), resuspended in ice-cold PBS with 0.1% BSA and filtered. This process was repeated four times, resulting in four fractions. Cells from each fraction were centrifuged, washed, and resuspended in ice-cold DMEM/F12 supplemented with glutamine and HEPES 15 mM. Each fraction was mixed with Geltrex GFR LDEV-free (Gibco, Thermo Fisher Scientific, USA) and IntestiCult™ Organoid Grow Medium Mouse (Stemcell Technologies™, Canada), added to a 24-well plate and incubated at 37 °C for 10 min for matrix polymerization. The organoids were cultured in IntestiCult™ Organoid Grow Medium Mouse, with medium changes every 2 days and splitting after 7 days.

For intestinal tolerability assays, selected hit compounds were tested in mouse intestinal organoids at 25 and 50 µM final concentrations. The protocol, based on a previous work [27], involved the addition of Gentle Cell Dissociation Reagent (Stemcell Technologies™, Canada) on matrix domes, the disruption of the matrix, and the incubation the suspension. After centrifugation, cells were resuspended in DMEM/F12 supplemented with glutamine and HEPES 15 mM and centrifugated again. The pellet was resuspended in a mix of Geltrex GFR LDEV-free and IntestiCult™ Organoid Grow Medium Mouse. The mix was plated in 384-well plates, and after 4 days, mature organoids were exposed to the compounds. The positive control was hydrogen peroxide (0.15% v/v), and the negative control was DMSO (0.2%). Viability after 72 h was assessed using the alamarBlue assay.

### In silico predictions: druglikeness and toxicity risks predictions

The potential toxicity and druggability of one of the most active compounds in terms of ovicidal and adulticidal activity (BZD31) were analyzed using the SwissADME and preADMET website platforms. The other selected compounds (BZD46, BZD56 and BZD59) were analyzed in a previous work [21].

The empirical formula and the corresponding unequivocal Simplified Molecular Input Line Entry System (SMILES) were introduced in the websites to obtain its predictive compliance with the “Lipinski Rule of 5” and ADMET parameters (including predictive mutagenicity and carcinogenicity).

### Data analysis

For the AVT, statistical analysis was performed using GraphPad Prism version 8.0.2. The normality of the motility inhibition scores was assessed using the Shapiro–Wilk test. Then, differences among scores at the three time points (24, 48, and 72 h) were evaluated using the Friedman test. In cases where significant differences were observed, post hoc analysis was conducted using the Nemenyi test for multiple comparisons. Comparisons with a *P* value < 0.05 were considered statistically significant.

To calculate the 50% cytotoxic concentration (CC_50_) value of each compound on cytotoxicity assays, cell viability expressed as the fluorescence emitted by resorufin at 590 nm was plotted against the corresponding concentration added to cell culture and fitted using the software package for scientific data analysis SigmaPlot 10.0 (Systat Software, Inc., San José, California, USA). The data are represented as mean ± standard error of media (SEM).

## Results

### Characterization of susceptible and resistant isolates

To carry out compound screening assays on eggs, two isolates from naturally infected cattle were specifically selected. In these isolates, the ovicidal activity of ABZ at a concentration of 0.5 µM reached 99.1%, indicating a high susceptibility.

In the case of the resistant isolate, ABZ-resistance was confirmed after showing a 96.7% survival rate of the adult flukes at the necropsy and after 2 weeks of ABZ treatment. Moreover, the reduction in the number of eggs in feces was 0%.

### Egg hatch test

The first screening performed at a dose of 50 µM (Tables 1 and 2) showed a total of 13 compounds with ovicidal activities higher than 80%. A total of 3 of these compounds belong to type I (BZD-04, BZD-08, and BZD20) and 11 to type II (BZD-27, BZD-30, BZD-31, BZD-32, BZD-38, BZD-46, BZD-55, BZD-56, BZD-58, BZD-59, and BZD-61) BZDs.

**Table 1:** Ovicidal activity of type I BZDs on an ABZ-susceptible isolate of *Fasciola hepatica* at a concentration of 50 µM


R_1_	R_2_	Compound identification	% Ovicidal activity
H	4′-OMe	BZD01	20.07 ± 9.32
H	4′-Cl	BZD02	09.55 ± 5.68
H	4′-Br	BZD03	01.93 ± 1.11
5-Me	4′-OMe	BZD04	99.57 ± 0.43
5-Me	4′-Cl	BZD05	35.59 ± 9.00
5-Me	2′,5′-diMe	BZD06	02.26 ± 1.30
5-Me	3′-NO_2_,4′-OMe	BZD07	07.13 ± 3.45
5-Cl	4′-OMe	BZD08	98.08 ± 1.06
5-Cl	4′-Cl	BZD09	04.96 ± 1.22
5-Cl	4′-NO_2_	BZD10	23.55 ± 8.06
5-Cl	2′,5′-diMe	BZD11	03.28 ± 1.45
5-Cl	3′-NO_2_,4′-OMe	BZD12	00.55 ± 9.45
5-Cl	3′-NH_2_,4′-OMe	BZD13	04.40 ± 1.38
5-NO_2_	4′-OMe	BZD14	15.55 ± 7.35
5-NO_2_	4′-Cl	BZD15	24.32 ± 10.58
5-NO_2_	2′,5′-diMe	BZD16	00.33 ± 0.33
5-NO_2_	3′-NO_2_,4′-OMe	BZD17	00.00 ± 0.00
5-NH_2_	4′-OMe	BZD18	02.82 ± 1.42
5-NHCO_Picol_	4′-OMe	BZD19	13.61 ± 3.64
5-OMe	4′-OMe	BZD20	87.02 ± 3.64
5-OMe	4′-Cl	BZD21	06.88 ± 6.88
6-OMe	4′-Br	BZD22	67.47 ± 5.64
5,6-diMe	4′-OMe	BZD23	59.08 ± 3.79
5,6-diCl	4′-OMe	BZD24	16.09 ± 6.50

Values are represented as a mean ± standard error of the mean (SEM)

Picol, picolinamide

**Table 2:** Ovicidal activity of type II BZDs on an ABZ-susceptible isolate of *Fasciola hepatica* at a concentration of 50 µM


X	R_1_	R_2_ or B	Compound identification	% Ovicidal activity
SO_2_	5-Me	4-Me	BZD25	3.94 ± 1.37
SO_2_	5-Me	B, naphth-2-yl	BZD26	2.23 ± 0.99
CH_2_	5-Me	H	BZD27	95.92 ± 1.99
CH_2_	5-Me	2,3,4-triOMe	BZD28	01.02 ± 0.62
CH_2_	5-Me	B, naphth-2-yl	BZD29	04.65 ± 1.98
CH_2_	5-Me	B, furan-2-yl	BZD30	100.00 ± 0.00
CH_2_	5-Me	B, thiofen-2-yl	BZD31	100.00 ± 0.00
CH_2_	5-Cl	H	BZD32	92.62 ± 1.96
CH_2_	5-Cl	4(OMe)	BZD33	05.52 ± 2.49
CH_2_	5-Cl	3,4,5-triOMe	BZD34	00.39 ± 0.38
CH_2_	5-Cl	B, furan-2-yl	BZD35	37.65 ± 13.55
CH_2_	5-OMe	H	BZD36	70.94 ± 2.32
CH_2_	5-OMe	4-OMe	BZD37	08.66 ± 1.86
CH_2_	5-OMe	B, 4-(pyrrolidin-1-yl)phenyl	BZD38	04.30 ± 2.79
CH_2_	5-OMe	B, 5-methylfuran-2-yl	BZD38	92.36 ± 2.43
CH_2_	5-OMe	B, thiofen-2-yl	BZD40	17.73 ± 5.12
CH_2_	5,6-diMe	4-OMe	BZD41	05.76 ± 3.12
CH_2_	5,6-diMe	3,4,5-triOMe	BZD42	10.73 ± 3.26
CH_2_	5,6-diCl	H	BZD43	55.36 ± 3.12
CH_2_	5,6-diCl	4-OMe	BZD44	05.99 ± 3,84
CH_2_	5,6-diCl	3,4,5-triOMe	BZD45	00.91 ± 0.61
CO	5-Me	H	BZD46	98.59 ± 0.94
CO	5-Me	B, 3-NO_2_-benzyl	BZD47	05.89 ± 2.62
CO	5-Me	4-OMe	BZD48	09.72 ± 2.50
CO	5-Me	B, 2-phenyl-2-hydroxymethyl	BZD49	10.56 ± 3.96
CO	5-Me	2-Cl,5-NO_2_	BZD50	07.62 ± 2.71
CO	5-Me	3,5-diOMe	BZD51	05.05 ± 2.51
CO	5-Me	3,4,5-triOMe	BZD52	02.50 ± 1.26
CO	5-Me	B, naphthyl-2-ylmethyl	BZD53	01.07 ± 0.62
CO	5-Me	B, thiophen-2-ylmethyl	BZD54	07.20 ± 3.47
CO	5-Me	B, pyridin-2-yl	BZD55	89.71 ± 3.87
CO	5-Cl	H	BZD56	99.58 ± 0.22
CO	5-Cl	4-OMe	BZD57	60.39 ± 10.70
CO	5-Cl	B, pyridin-2-yl	BZD58	98. 85 ± 5.12
CO	5-OMe	H	BZD59	100.00 ± 0.00
CO	5-OMe	4-OMe	BZD60	01.08 ± 0.79
CO	5-OMe	B, pyridin-2-yl	BZD61	93.17 ± 3.31
CO	5-OMe	B, (boc)αα^*a*^	BZD62	02.95 ± 2.57
CO	5-OMe	B, αα^b^	BZD63	00.00 ± 0.00
CO	5,6-diMe	Phenyl	BZD64	04.51 ± 2.57
CO	5,6-diMe	3-Cl	BZD65	01.61 ± 0.82
CO	5,6-diMe	4-OMe	BZD66	00.26 ± 0.25
CO	5,6-diCl	H	BZD67	03.33 ± 2.51
CO	5,6-diCl	3-NO_2_benzyl	BZD06	04.63 ± 2.27
CO	5,6-diCl	3-NH_2_benzyl	BZD69	03.90 ± 2.68
CO	5,6-diCl	4-OMe	BZD70	02.75 ± 1.16

Values are represented as a mean ± standard error of the mean (SEM)

^a^1-Boc-aminoundecyl; ^b^aminoundecyl

A further screening was then carried out with these 13 selected compounds at a dose of 10 µM (Table 3), showing that four type II BZDs (BZD-31, BZD-46, BZD-56, BZD-59) reached the established threshold value of 80%. Their ovicidal activity ranged between 83.9% for BZD-46 to 99.5 for BDZ-31. Whereas the activity of BZD-05, BZD-08, BZD-27, BZD-30 and BZD-58 decreased to values around 50% at this concentration.

**Table 3:** Ovicidal activity of BZDs on an ABZ-susceptible isolate of *Fasciola hepatica* at a concentration of 10 µM along with cytotoxicity values on HepG2 cells and toxicity assays carried out with organoids

Compound identification	% Ovicidal activity	CC_50_	% Viability in organoids	% Viability in organoids
	10 µM	Hep-G2	25 µM	50 µM
BZD04	64.27 ± 10.16	10.78 µM	21.10%	10.50%
BZD08	55.33 ± 05.81	> 50 µM	69.30%	47.30%
BZD20	24.59 ± 05.71	–	–	–
BZD27	44.65 ± 07.46	20.29 µM	54.10%	49.70%
BZD30	52.41 ± 01.02	> 50 µM	62.50%	54.00%
BZD31	99.58 ± 00.26	> 50 µM	60.50%	52.40%
BZD32	09.11 ± 03.40	37.41 µM	48.10%	52.10%
BZD38	03.23 ± 00.84	> 100 µM	81.10%	38.00%
BZD46	83.90 ± 02.87	> 100 µM	99.70%	87.40%
BZD55	07.11 ± 03.01	> 100 µM	98.60%	100%
BZD56	93.93 ± 01.90	> 100 µM	82.50%	95.90%
BZD58	45.12 ± 07.23	> 5 µM	100%	01.50%
BZD59	91.16 ± 02.58	> 100 µM	82.70%	56.70%
TCBZ	–	> 50 µM	81.00%	01.60%
ABZ (DD)	99.01 ± 00.37	–	–	–

Values are represented as a mean ± standard error of the mean (SEM)

ABZ, albendazole; CC_50_, cytotoxic concentration 50; DD, discriminant dose to detect ABZ resistance; TCBZ, triclabendazole

**Fig. 1:**
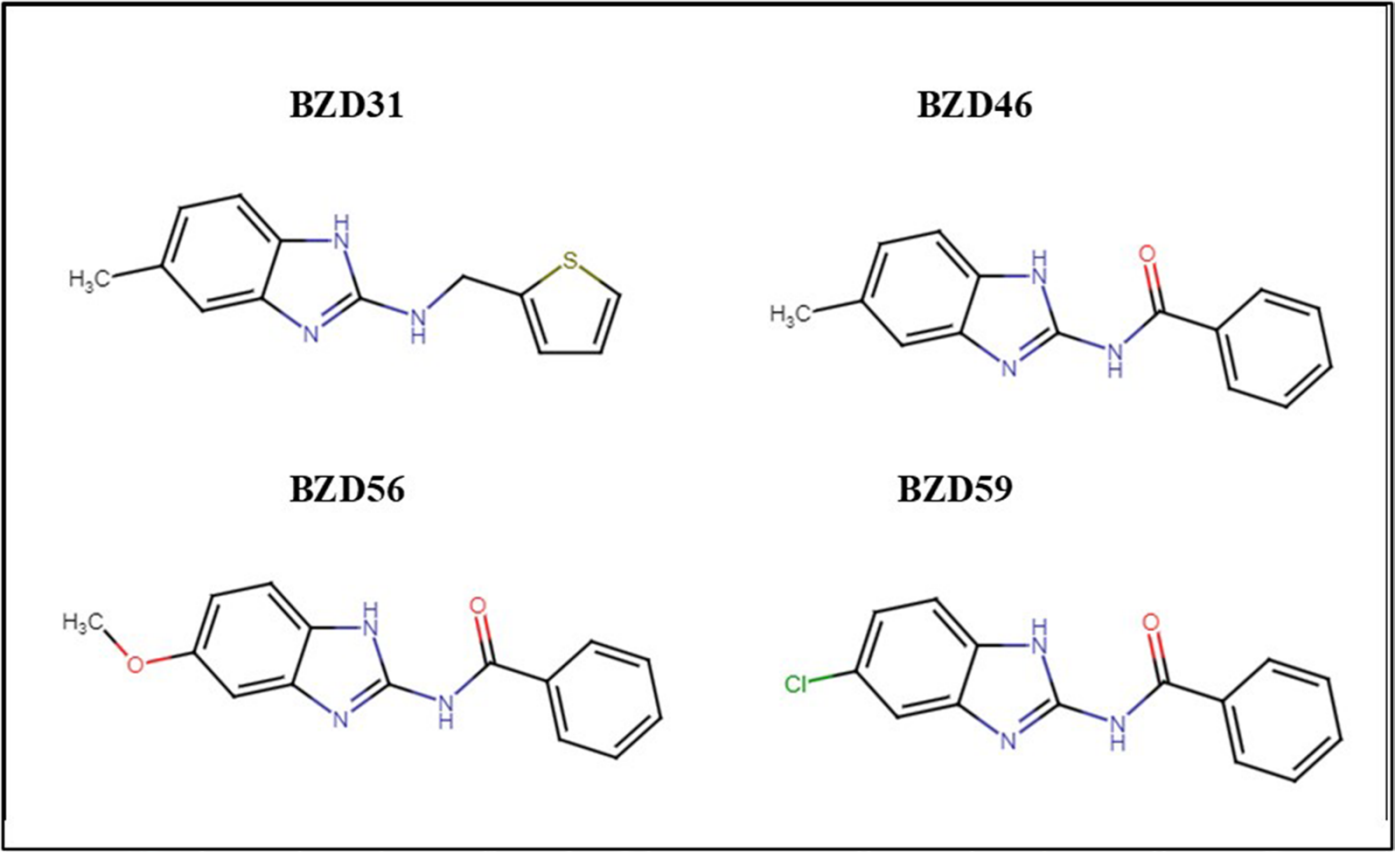
Chemical structure of the hit compounds identified in the present work

In terms of the screening performed at 5 µM (Table 4) with the most effective BZDs (Fig. 1), all of them remained highly active displaying ovicidal activities higher than 71%. Three commercial BZ drugs were also tested against eggs at the same concentration. OXF and the ABZ metabolite ABZSO showed activities of over 98%. All these selected compounds and drugs were tested in eggs from the resistant isolate, showing a clear reduction in their activity for most BZDs (BZD-46, BZD-56, and BZD-59) to values below 13%. However, BDZ31 still displayed a significant ovicidal activity of 53% at this concentration in the resistant isolate, in comparison with an activity of 74% in the susceptible one. The same pattern was observed for OXF and ABZSO, both with an important loss of activity in this resistant isolate, from 98% and 99% in the susceptible isolate, to 3% and 26%, in the resistant one, respectively.

**Table 4:** Ovicidal activity of BZDs, CLR, TCBZ, ABZSO, and OXF at a dose of 5 µM against *Fasciola hepatica*, susceptible and resistant to ABZ

Compound identification	% Ovicidal activity susceptible isolate	% Ovicidal activity resistant isolate
BZD31	74.59 ± 05.72	53.24 ± 20.94
BZD46	71.21 ± 09.91	11.06 ± 05.30
BZD56	89.22 ± 02.88	12.39 ± 03.71
BZD59	73.51 ± 08.88	06.28 ± 05.20
OXF	98.48 ± 00.37	03.41 ± 02.08
ABZSO	99.37 ± 00.34	26.12 ± 11.87
TCBZ	01.60 ± 01.44	–
CLR	00.70 ± 04.50	–
ABZ (DD)	98.55 ± 00.41	36.46 ± 02.36

Values are represented as a mean ± standard error of the mean (SEM). All experiments were done by triplicate two different days

ABZ, albendazole; ABZSO, albendazole sulfoxide; CLR, clorsulon; DD, discriminant dose to detect ABZ resistance; OFX, oxfendazole; TCBZ, triclabendazole

### Adult viability test

**Fig. 2:**
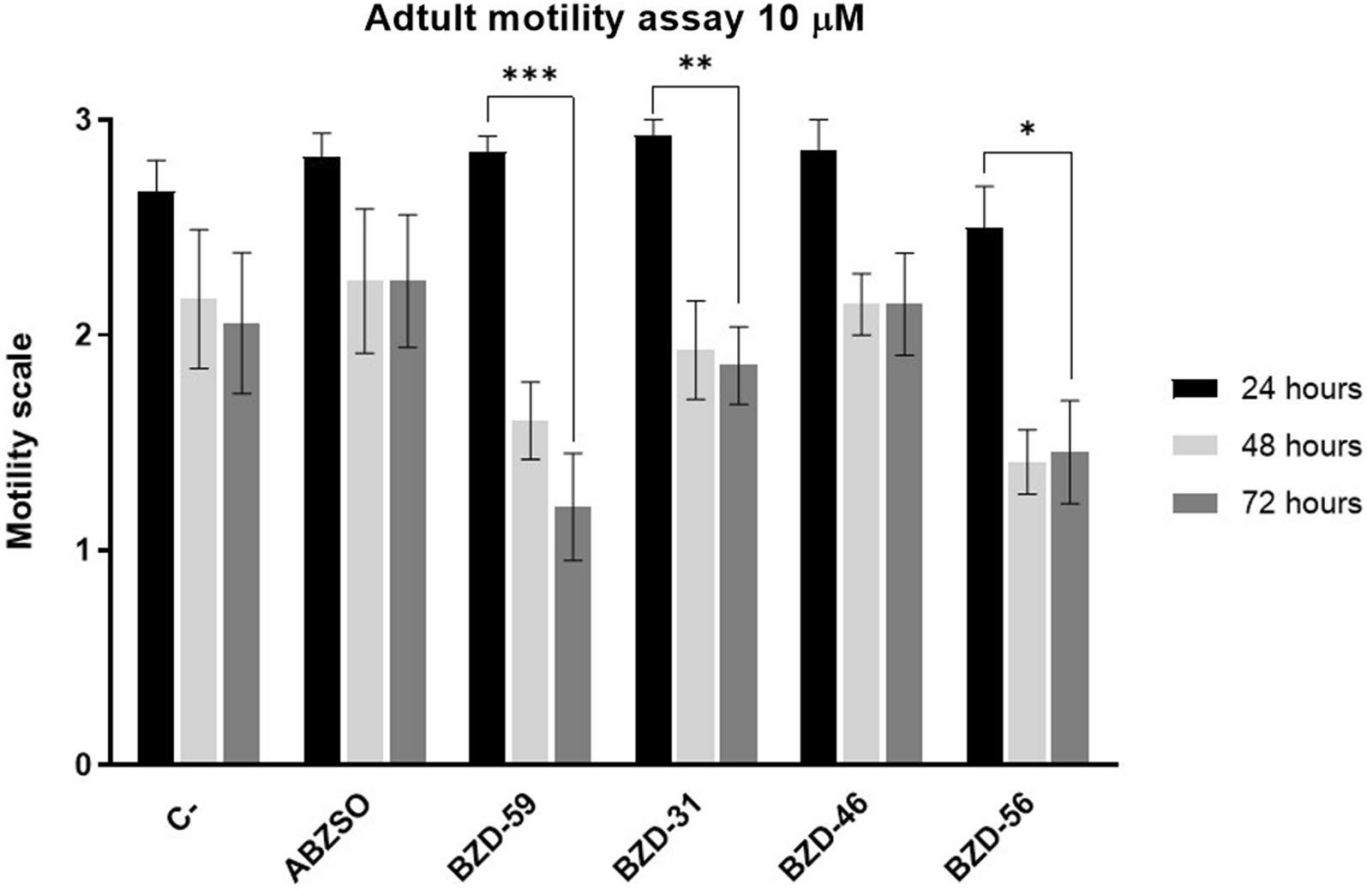
Motility inhibition of BZDs and ABZSO at a dose of 10 µM on *Fasciola hepatica* adults from an ABZ-resistant isolate at three different time points (24, 48, and 72 h) after treatment. All experiments were done at least by triplicate and two different days. C− represents the negative control. The data are represented as mean ± standard error of mean (SEM). *** (*P* < 0.001), ** (*P* < 0.005), * (*P* < 0.05)

The four selected compounds (BZD31, BZD46, BZD56, and BZD59) and ABZSO were also evaluated in the adult stage of a resistant isolate at a final concentration of 10 µM (Fig. 2). The statistical analysis of their activity revealed that BZD31 (*P* < 0.005), BZD56 (*P* < 0.01), and BZD59 (*P* < 0.001) showed a significant reduction of their activities after 72 h of incubation compared to the first time point measurement at 24 h. Interestingly, neither the negative wells, containing only DMSO, nor those with ABZSO or BZD46, exhibited any significant motility inhibition after 72 h. Remarkably, after 72 h of incubation, BZD59 produced the most significant reduction (Table 5) in parasite motility among all compounds (60%), even more than ABZSO, which did induce a motility inhibition of 25% at the same concentration.

**Table 5:** Percent of motility inhibition induced by BZDs and ABZSO at a dose of 10 µM on an ABZ-resistant isolate of *Fasciola hepatica* adults after 48 and 72 h of treatment

Compound	% Adult motility inhibition 48 h	% Adult motility inhibition 72 h
C− (DMSO 0.5%)	27.8	31.5
ABZO	25.0	25.0
BZD31	35.7	38.1
BZD46	28.6	28.6
BZD56	53.0	51.5
BZD59	46.7	60.0

ABZ, albendazole; ABZO, albendazole sulfoxide; C−, negative control

### Cytotoxicity and intestinal tolerability assays

The cytotoxicity of 12 compounds and TCBZ for the HepG2 cell line were evaluated (Table 3). The 50% cytotoxic concentration (CC_50_) values were above 50 μM for all compounds except for BZD04, BZD27, BZD32, and BZD58, which showed significative toxicity (CC_50_ > 5 μM).

In terms of intestine tolerability, three compounds (BZD46, BZD56, and BZD55) showed viabilities over 87% when exposed at 50 μM, while two displayed values of around 1.5% (BZD58 and TCBZ). The rest of the BZDs had viability values between 38% and 56%. When incubating at 25 μM, it was observed a considerable increase of viability for all compounds with values ranging from 48% to 100%, with the exception of BZD04 that showed 21% of viability.

### In silico predictions: druglikeness and toxicity risks predictions

Additional file 1: Table S1 shows the predictive druggability and toxicity results of BZD31 based on its structure. As mentioned before, the potential toxicity and druggability data of compounds BZD46, BZD56, and BZD59 were obtained from a previous study [21].

According to data displayed in Table S1, BZD31 complies with Lipinsky’s rule of five for possible oral administration as it is a small molecule (molecular weight < 500 Da) and has less than five hydrogen-bond donors (HBD) and less than ten hydrogen-bond acceptors (HBA). In addition, the prediction of gastrointestinal absorbability is high. However, BZD31 showed values indicative of probable blood–brain barrier crossing. Predictions regarding the potential of this compound as substrate and/or inhibitor of the P-glycoprotein (P-gp)—an important member of ATP-binding cassette transporter family—reveal that this compound is not a substrate of this P-gp but not an inhibitor. Additional file 1: Table S1 also shows the predictive metabolization by a group of human cytochromes P450 (CYP) involved in oxidative processes in the cell. According to both online platforms, this compound could be a potent substrate of CYP2D6 and weakly of CYP3A4; it also seems to be a potent inhibitor of CYP1A2, CYPAC19, CYP2C9, and CYP2D6 but not of CYP3A4, which could lead to its accumulation within the organism and then to toxicity.

Toxicity risks predictions, including mutagenicity via the Ames test and rodent carcinogenicity, showed that BZD31 has a moderate probability of being mutagenic and carcinogenic. The introduction of TCBZ structure on the preADMET platform similarly revealed mutagenic and carcinogenic properties (Additional file 1: Table S2). On the other hand, toxicity predictions calculated for the other three selected BZD (BZD46, BZD56 and BZD59) suggested that all of them are safe [21]. They also comply with Lipinsky’s rule of five, showing positive druglikeness and leadlikeness qualifications.

## Discussion

An increasing number of treatment failures have been reported worldwide in livestock animals infected with *Fasciola* spp. [14, 16, 28–31]. In recent decades, the same pattern has been observed in humans, with reported cases spread across different countries, such as Peru, Chile, the Netherlands, Portugal, or Turkey [18, 19, 32–35]. In view of the increasing threat of anthelmintic resistance to all currently used formulations, attention should be focused on the development of new fasciolicidal compounds and vaccines [36–40].

Drug discovery is a complex process that encompasses different approaches. While the synthesis of new chemical entities stands out as the most efficient method, it is a really time-consuming and cost-intensive process [41]. Consequently, alternative strategies come to the forefront, offering new paths for exploration. Among these, the synthesis of derivatives from known drugs with approved usage, or the modification of their structures to generate new compounds with enhanced properties and effectiveness (me-too drugs), could play a key role in the drug discovery process [42]. In fact, this approach can lead to the development of more potent compounds with enhanced solubility and pharmacokinetic properties compared with those discovered thus far. Examples of successful outcomes include the BZD mebendazole nitrate [43], the macrocyclic lactones tenvermectin [44] and moxidectin [45], as well as the imidazole derivative diisopropylphenyl-imidazol [46], all developed for the treatment of gastrointestinal nematodes. Nevertheless, the use of drugs derived from the same family of compounds presents a substantial risk of anthelmintic resistance emergence [47]. However, exceptions exist, as evidenced by reported cases of ABZ-resistant but TCBZ-susceptible *F. hepatica*, documented in countries such as Argentina and Sweden [48–50]. In support of these findings, our research group has recently described a field isolate of *F. hepatica* susceptible to TCBZ but resistant to ABZ in sheep (unpublished data).

The EHT was initially developed by [25] as a method for detecting the presence of *F. hepatica* isolates resistant to BZs [51, 52]. However, this technique has proven to be an efficient approach for screening a large number of compounds against *Fasciola* eggs, and it has been adopted by several researchers in the field [39, 53].

Under this context, the potential fasciolicidal activity of various series of new BZDs was assessed along this study by means of two different in vitro assays, the EHT and the AVT. Type I BZDs were first screened in a previous study against two different isolates of the gastrointestinal nematode *Teladorsagia circumcincta*, one susceptible isolate and another resistant to BZs. The results of that study showed that BZD9 was the most effective compound, displaying a 50% inhibitory concentration (IC_50_) value of 6 µM in eggs by the EHT [20]. Some of these compounds were also evaluated in two rodent models of other gastrointestinal nematodes, demonstrating that BZD09 effectively inhibited the motility of *Heligmosomoides polygyrus* adults at a concentration of 10 µM, while BZD15 inhibited *Trichuris muris* adult motility at the same concentration [54]. However, in the current study, none of these compounds exhibited activities exceeding 25% when tested against *F. hepatica* eggs at a concentration of 50 µM. Based on these findings, new BZDs (type II) with modified structures were synthesized, leading to a novel series of BZDs compounds with enhanced properties [21]. These newer series achieved IC_50_ values around 1 µM against *T. circumcincta* eggs (BZD46, BZD55, BZD56, and BZD59). Notably, BZD61 showed an IC_50_ below 1 µM and a selective index over 100 in HepG2 cells, being the most active and safest compound. Three out of these four derivatives (BZD46, BZD56, and BZD59) were also the most active compounds in the present study against *F. hepatica*. Another study reported that BZD58 (the 2-arylcarboxamido type II), administered at a dose of 120 mg/kg, exhibited in vivo efficacy of 95% in sheep infected with the gastrointestinal nematode *Haemonchus contortus* [22]. However, this compound displayed an ovicidal effect of less than 50% in *Fasciola* at a dose of 10 µM. Taken together, these results clearly affirm that structural modifications introduced to these type II derivatives led to molecules with improved activity.

With respect to the screenings carried out at 50 and 10 µM, four hit compounds (the 2-arylmethylamino BZD31 and the 2-arylcarboxamido BZD46, BZD56, and BZD59) classified under type II BZDs (Fig. 2) progressed to subsequent assays. At this stage, their activity was evaluated alongside commercial drugs, three BZs (TCBZ, OXF, and ABZSO) and CLR, at a concentration of 5 µM, to compare their activities. While OXF has demonstrated in vitro and in vivo properties, it is not currently a recommended flukicide, in contrast to ABZSO, TCBZ, and CLR [55, 56]. When performing the EHT on a susceptible *F. hepatica* isolate at 5 µM, all hit compounds showed activities exceeding 71%, with BZD56 being the most potent one, displaying an activity slightly below 90%. OXF and ABZSO reached values around 99% at the same concentration, while TCBZ and CLR did not overcome 2%. These results align with other studies, which showed that TCBZ and CLR did not affect in vitro egg hatching, whereas ABZ is able to reduce egg hatching at concentrations as low as 0.05 µM. In contrast, ABZSO and OXF required higher concentrations to achieve comparable activity, also in accordance with a previous study [56]. Therefore, while all hit compounds displayed high activity at 5 µM, none reached the activity of ABZSO, ABZ, or OXF at the same concentration.

When evaluating the efficacy of commercial compounds on the *F. hepatica* resistant isolate at 10 µM, OXF and ABZSO activity decreased to values of 3.5% and 26%, respectively, indicative of a high level of side-resistance between BZs. Furthermore, ABZ activity at 0.5 µM, the concentration used as DD to differentiate susceptible and resistant isolates [25], dropped from 98% in the susceptible isolate to 36.5% in the resistant one, confirming again the presence of ABZ-resistance. In addition, all BZDs showed a significant reduction in their ovicidal activities. The activity of BZD59, BZD46, and BZD56 dropped to values below 13%, while BZD31 remained considerable active, with an ovicidal activity close to 53%. BZD31 shows a methylene between the aryl fragment and the BZ ring, instead of a carbonyl as in BZD56. As we indicated in Escala et al. [21], the presence of an amine in position 2 (BZD type II) allows the existence of different tautomeric equilibria, which will be different in the 2-arylmethylamino BZD31 with respect to 2-arylcarboxamido BZD56. Moreover, BZD31 shows a heterocycle (thiophene) with respect to a phenyl ring in BZD56. In this sense, compound BZD31 could present some tautomeric forms in its interaction with the target but different from those observed in the amide-type BZDs (BZD46 to BZD70), suggesting a different mode of action of this compound with respect to the others.

As regards the AVT, BZD59 was the most potent compound after 72 h of incubation, while ABZSO showed no evidence of inhibition after this period of time. Therefore, we conclude that both BZD31 and BZD59 were the most active compounds according to the in vitro experiments conducted on *F. hepatica* eggs and adults, respectively, as they achieved higher activities than the marketed compounds when screened against the resistant isolate.

Cytotoxicity data discarded the possible toxicity of BZD31 and BZD56 on HepG2 cells as both showed CC_50_ values higher than 50 and 100 µM, respectively. Data from a previous work conducted by our research group also demonstrated that BZD59 is not toxic when incubating on Caco2 cells, as it displayed a CC_50_ value of around 62 µM [21]. In terms of small intestine toxicity, both compounds suggest a well tolerability at high concentration (50 µM) as the percentage of viability was 56.7 and 95.9, in contrast to TCBZ, which was only 1.5%. After reducing the concentration by half, intestine viability values of both compounds and TCBZ were around 82%.

Previously ADMET predictions performed on BZD59 through the web services SwissADME and ADMETsar confirmed its positive druglikeness and leadlikeness qualification, while predictions of mutagenic, tumorigenic and irritant toxicity risk were discarded [21], thus confirming the potential of this compound as a starting point for the development of a fasciolicidal compound. Druggability predictions for BZD31 were positive as in the case of BZD56, whereas it showed to have possible mutagenic and carcinogenic properties in rat and mouse (Additional file 1: Table S1).

In terms of mechanism of action of BZ family, it is established that TCBZ and others compounds such as ABZ and mebendazole disturb the secretory processes of the fluke. Specifically, TCBZ causes the disruption of the tegument of *F. hepatica*, and both TCBZ and ABZ inflict severe damage to the reproductive system [57]. While the primary mode of action of TCBZ is suspected to involve the disruption of microtubule-based processes [58–60], the exact binding site remains unknown [61, 62]. Thus, the primary mode of action of TCBZ metabolites in *F. hepatica* is still inconclusive, as they have the potential to interact with various biological systems, exerting cascading effects that have yet to be fully explored [63]. Therefore, we hypothesize that our new compounds could share a similar mechanism of action, though not necessarily identical, while maintaining a wide safety margin in alignment with the BZ family [64]. This opens up the possibility of discovering other potent derivatives. Nevertheless, further studies are required to comprehensively explore these hypotheses.

The challenges and the scarcity of studies on the screening of compounds with potential flukicidal activity reinforce the significance of the present work, advancing the search for new compounds with fasciolicidal activity [39, 40, 53, 65]. However, further investigations are necessary to determine the in vitro activity of the compounds against juvenile worms, as well as in vivo toxicity assays, pharmacokinetic profile analysis, and in vivo efficacy determination.

## Conclusions

BZD56 was found as the most effective compound, displaying an ovicidal activity of 89% against a susceptible isolate of the parasite at a concentration of 5 μM. On the other hand, BZD31 achieved the highest ovicidal acitivity (53%) when tested against an ABZ-resistant isolate. The efficacy of ABZSO and OXF at the same concentration decreased from 99% and 98% to 26% and 3%, respectively, in the resistant isolate. When these compounds were screened against resistant adult worms, BZD59 showed the most significant reduction in worm motility (p < 0.001) after 72 h of incubation, while ABZSO did not produce any motility alteration, confirming the ABZ resistance. Both BZDs seemed to be safe when cultured on HepG2 and intestinal organoids, suggesting that they could be promising candidates for further in vivo trials or as a starting point for the new synthesis of structure-related compounds.

### Supplementary Information

**Additional file 1: Table S1.** Virtual absorption, distribution, metabolism, excretion (ADME) predictions of BZD31 by SwissADME website. **Table S2.** Virtual absorption, distribution, metabolism, excretion and toxicity (ADMET) predictions of BZD31 obtained in preADMET website.

## Data Availability

The datasets supporting the conclusions of this article are included within the article text and additional files.

## Abbreviations

ABZ: Albendazole
ABZSO: Albendazole sulfoxide
ADMET: Absorption, distribution, metabolism, excretion, and toxicity
AVT: Adult viability test
BZ: Benzimidazole
CC_50_: Cytotoxic concentration 50
CYP: Cytochromes P450
DD: Discriminant dose
DMSO: Dimethyl sulfoxide
EHT: Egg hatching test
EPG: Egg per grams of feces
FECRT: Fecal egg count reduction test
HBA: Hydrogen-bond acceptors
HBD: Hydrogen-bond donors
IC_50_: Inhibitory concentration 50
OXF: Oxfendazole
PAHO: Pan American Health Organization
SEM: Standard error of the median
SI: Selectivity index
TCBZ: Triclabendazole
