# Supplementary material for: New benzimidazole derivative compounds with in vitro fasciolicidal properties
Source: Parasit Vectors. 2024 Apr 3;17:173. doi: 10.1186/s13071-024-06224-6 (PMC10993450; doi:10.1186/s13071-024-06224-6)
Supplement: Supplementary file 1 — Additional file 1: Table S1. Virtual absorption, distribution, metabolism, excretion (ADME) predictions of BZD31 by SwissADME website. Table S2. Virtual absorption, distribution, metabolism, excretion and toxicity (ADMET) predictions of BZD31 obtained in preADMET website. [file 13071_2024_6224_MOESM1_ESM.docx]

**BZD31**


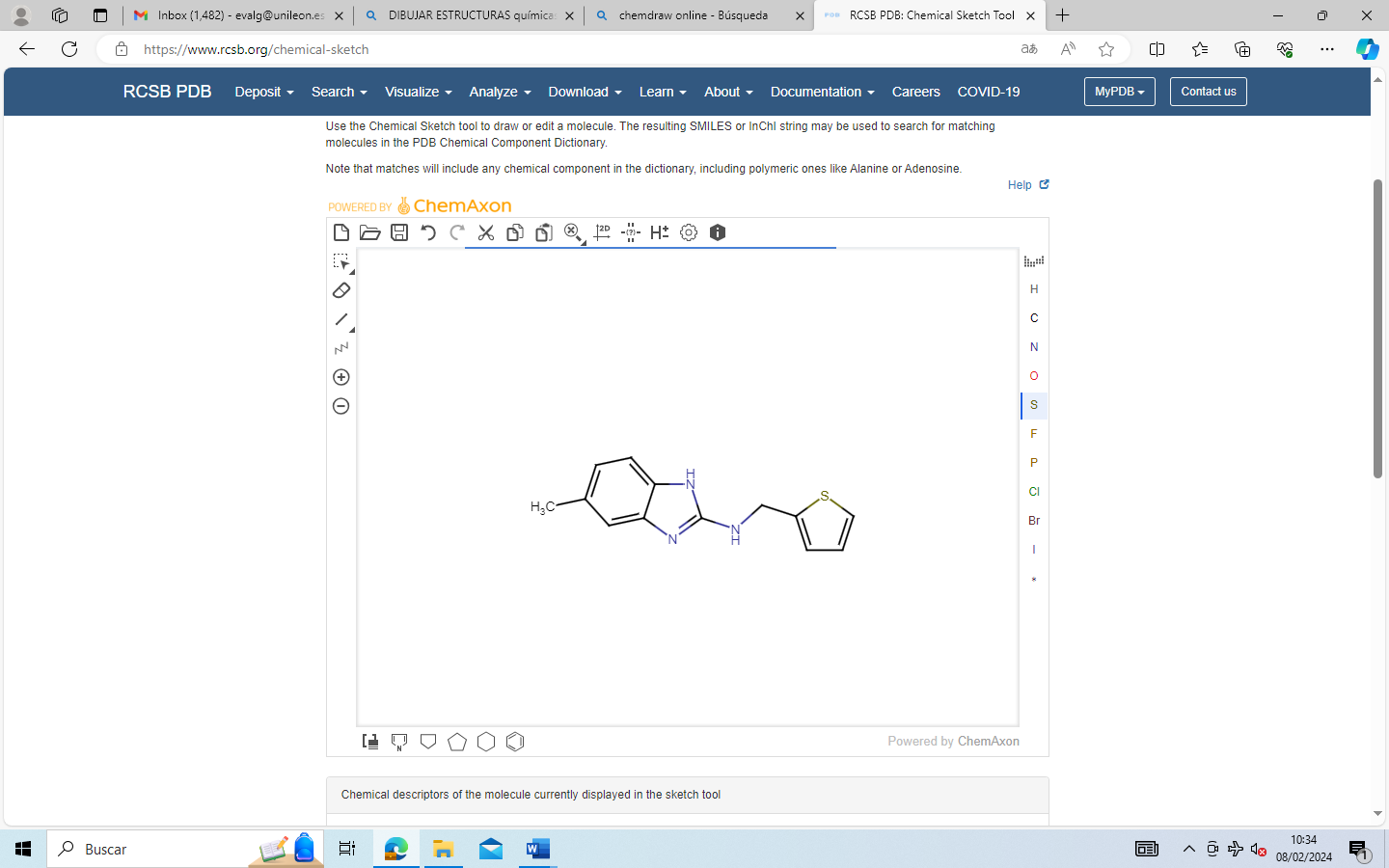


| **SMILES** | Cc1ccc2c(c1)nc([nH]2)NCc1cccs1 |
| --- | --- |

| **Physicochemical Properties** | | | |
| --- | --- | --- | --- |
| **Formula** | C13H13N3S | **Num. rotatable bonds** | 3 |
| **Molecular weight** | 243.33 g/mol | **Num. H-bond acceptors** | 1 |
| **Num. heavy atoms** | 17 | **Num. H-bond donors** | 2 |
| **Num. arom. heavy atoms** | 14 | **Molar Refractivity** | 72.73 |
| **Fraction Csp3** | 0.15 |  |  |
|  |  |  |  |
| **Lipophilicity** | | | |
| **Log Po/w (iLOGP)** | 2.08 | **Log Po/w (MLOGP)** | 2.37 |
| **Log Po/w (XLOGP3)** | 2.99 | **Log Po/w (SILICOS-IT)** | 4.24 |
| **Log Po/w (WLOGP)** | 3.20 | **Consensus Log Po/w** | 2.98 |
|  |  |  |  |
| **Water Solubility** | | | |
| **Log S (ESOL)** | -3.64 | **Class** | Moderately soluble |
| **Solubility** | 5.53e-02 mg/ml  2.27e-04 mol/l | **Log S (SILICOS-IT)** | -5.55 |
| **Class** | Soluble | **Solubility** | 6.91e-04 mg/ml ; 2.84e-06 mol/l |
| **Log S (Ali)** | -4.10 | **Class** | Moderately soluble |
| **Solubility** | 1.92e-02 mg/ml  7.91e-05 mol/l |  |  |
|  |  |  |  |
| **Pharmacokinetics** | | | |
| **GI absorption** | High | **CYP2C9 inhibitor** | No |
| **BBB permeant** | Yes | **CYP2D6 inhibitor** | Yes |
| **P-gp substrate** | Yes | **CYP3A4 inhibitor** | Yes |
| **CYP1A2 inhibitor** | Yes | **Log Kp (skin permeation)** | -5.66 cm/s |
| **CYP2C19 inhibitor** | Yes |  |  |
|  |  |  |  |
| **Druglikeness** | | | |
| **Lipinski** | Yes; 0 violation | **Egan** | Yes |
| **Ghose** | Yes | **Muegge** | Yes |
| **Veber** | Yes | **Bioavailability Score** | 0.55 |
|  |  |  |  |
| **Medicinal Chemistry** | | | |
| **PAINS** | 0 alert | **Leadlikeness** | No; 1 violation: MW<250 |
| **Brenk** | 0 alert | **Synthetic accessibility** | 2.15 |

**Table S1.** Virtual absorption, distribution, metabolism, excretion (ADME) predictions of BZD31 by SwissADME website.

**Toxicity**

| **Ames_test** | Mutagen | **minnow_at** | 0.006147 |
| --- | --- | --- | --- |
| **Carcino_Mouse** | Positive | **TA100_10RLI** | Positive |
| **Carcino_Rat** | Positive | **TA100_NA** | Negative |
| **daphnia_at** | 0.048329 | **TA1535_10RLI** | Negative |
| **hERG_inhibition** | Medium_risk | **TA1535_NA** | Positive |
| **medaka_at** | 0.004371 |  | |
|  |  |  | |
| **ADME** | | | |
| **BBB** | 2.41987 | **Plasma_Protein_Binding** | 82.840323 |
| **CaCo2** | 23.3646 | **Pure_water_solubility_mg_L** | 8.846640 |
| **CYP_2C19_inhibition** | Non | **Skin_Permeability** | -4.063900 |
| **CYP_2D6_inhibition** | Non | **SKlogD_value** | 3.721930 |
| **CYP_2D6_substrate** | Non | **SKlogP_value** | 3.721039 |
| **CYP_3A4_inhibition** | Non | **SKlogS_buffer** | -3.345990 |
| **CYP_3A4_substrate** | Weakly | **logS_pure** | -4.439410 |
| **Pgp_inhibition** | Non |  | |

**Table S2.** Virtual absorption, distribution, metabolism, excretion and toxicity (ADMET) predictions of BZD31 obtained in preADMET website.
